# Surveillance of Wastewater to Monitor the Prevalence of Gastroenteritis Viruses in Chiba Prefecture (2014–2019)

**DOI:** 10.2188/jea.JE20220305

**Published:** 2024-04-05

**Authors:** Chiemi Hotta, Yuki Fujinuma, Takashi Ogawa, Mamiko Akita, Tomoko Ogawa

**Affiliations:** Division of Virology and Medical Zoology, Chiba Prefectural Institute of Public Health, Chiba, Japan

**Keywords:** wastewater-based epidemiology, surveillance, gastroenteritis, virus, sewage

## Abstract

**Background:**

In Japan, sentinel surveillance is used to monitor the trend of infectious gastroenteritis. Another method of pathogen surveillance, wastewater-based epidemiology, has been used recently because it can help to monitor infectious disease without relying on patient data. Here, we aimed to determine the viral trends reflected in the number of reported patients and number of gastroenteritis virus-positive samples. We focused on gastroenteritis viruses present in wastewater and investigated the usefulness of wastewater surveillance for the surveillance of infectious gastroenteritis.

**Methods:**

Real-time polymerase chain reaction was used for viral gene detection in wastewater. The number of reported patients per pediatric sentinel site and number of viral genome copies were compared for correlation potential. The number of gastroenteritis virus-positive samples reported by National Epidemiological Surveillance of Infectious Disease (NESID) and the status of gastroenteritis viruses detected in wastewater were also evaluated.

**Results:**

Genes of norovirus genotype I, norovirus genotype II, sapovirus, astrovirus, rotavirus group A, and rotavirus group C were detected in wastewater samples. Viruses were detected in wastewater during periods when no gastroenteritis virus-positive samples were reported to NESID.

**Conclusion:**

Norovirus genotype II and other gastroenteritis viruses were detected in wastewater even during periods when no gastroenteritis virus-positive samples were found. Therefore, surveillance using wastewater can complement sentinel surveillance and is an effective tool for the surveillance of infectious gastroenteritis.

## INTRODUCTION

Acute gastroenteritis continues to be a significant cause of morbidity and mortality.^1^^–^^3^ The estimated economic loss due to acute gastroenteritis is huge; for example, the economic loss caused by norovirus has exceeded $60 billion.^4^ Infectious gastroenteritis has been a common disease affecting all age groups in developed countries.^1^^,^^2^ There are two major methods used for infectious disease surveillance: sentinel surveillance and population-based surveillance. Sentinel surveillance can provide disease and epidemic trends based on cases identified at participating medical facilities. By contrast, population-based surveillance can help to estimate the incidence of diseases; however, this method is more costly than sentinel surveillance.^5^ Sentinel surveillance has been adopted to monitor the trend of infectious gastroenteritis among pediatric patients in Japan^6^; however, this may not reveal the trend of infectious gastroenteritis in the entire community. Additionally, not all pediatric institutes participating in sentinel surveillance in Japan have contributed to the identification of the pathogens causing infectious gastroenteritis. According to a 2016 report, the number of stool samples submitted for pathogen identification decreased in approximately one-third of the surveyed institutions between 2014 and 2016.^7^ This reduction in sampling, and hence the opportunity to identify pathogens, potentially impacts the ability to determine infectious gastroenteritis trends.

After the onset of the coronavirus disease pandemic, another method of population-based pathogen surveillance, wastewater-based epidemiology (WBE), received increased attention. This method was used to detect the early stage of an epidemic in a certain area.^8^^,^^9^ WBE can also reveal the status of infected individuals who have not been diagnosed at a medical institution. Analyzing wastewater is very similar to collecting and analyzing local fecal samples.^10^ There are many reports of the detection of gastroenteritis-causing viruses in wastewater.^11^^–^^13^ A previous study conducted in a small area suggested the usefulness of WBE in monitoring norovirus (NoV) genotype II (GII) in the community.^14^ Because testing was limited to NoV, no other viral causes for gastroenteritis in the reported patients were identified. Therefore, the usefulness of wastewater in epidemiological studies to monitor other viruses causing gastroenteritis is unclear.

WBE is a surveillance with a different viewpoint from sentinel surveillance. In this study, we aimed to determine the usefulness of WBE in the surveillance of infectious gastroenteritis. For this purpose, we focused on six specific gastroenteritis-causing viruses and the number of gastroenteritis virus-positive specimens in wastewater over a 5-year period. Furthermore, we attempted to determine which viruses were associated with the number of reported patients per pediatric sentinel site of infectious gastroenteritis.

## METHODS

### Wastewater collection

Wastewater samples were collected monthly at one selected wastewater pumping station in Chiba Prefecture between April 2014 and December 2019. The population in the area covered by this pumping station was estimated to be approximately 411,000 as of January 1, 2019. Wastewater treatment in this station is based on a separate sewer system in which wastewater and rainwater are treated separately. Approximately 2 L of wastewater was collected on each sampling day.

### Detection of viral genes in wastewater samples

The concentration of wastewater samples was performed through a previously described method,^15^ with some modifications. Briefly, the collected samples were stored at 4°C for up to 1 week before the examination. Of the 2 L sample, 500 mL was centrifuged at 3,000 rpm (1,920 xg) for 30 min, and magnesium chloride was added to the supernatant to achieve a final concentration of 0.05 M. The pH was adjusted to 3.5 using 1.0 N hydrochloric acid, and virus adsorption was performed using pressure filtration on a negative charge membrane with a pore size of 0.45 µm (ADVANTEC, Tokyo, Japan). The adsorbed viruses were eluted using 10 mL of 3% beef extract solution. The final volume of the eluted sample was 10 mL.

Viral ribonucleic acid (RNA) was extracted from the eluted samples using MagNA Pure LC 2.0 (Roche Diagnostics, Basel, Switzerland), an automated nucleic acid extraction system, with a MagNa Pure LC Total Nucleic Acid Isolation Kit (Nippon Genetics, Tokyo, Japan). Complementary deoxyribonucleic acid (cDNA) from the extracted RNA was synthesized using a PrimeScript RT reagent kit (Perfect Real Time; Takara, Kyoto, Japan) with GeneAmp PCR System 9700 or SimpliAmp Thermal Cycler (Applied Biosystems, Foster City, CA, USA). Genes of NoV genotype I (NoV GI),^16^ NoV GII,^16^ sapovirus (SaV),^17^ astrovirus (AstV),^18^ group A rotavirus (RVA),^19^ and group C rotavirus (RVC) virus^20^ were detected through real-time polymerase chain reaction (PCR) performed with Applied Biosystems Real-Time PCR Systems 7000, 7500, 7500 Fast, or StepOnePlus (Applied Biosystems). Commonly detected gastroenteritis viruses were targeted.^2^^,^^3^ The number of NoV GI, NoV GII, and SaV genome copies in the concentrated samples was determined using a standard curve generated from a 10-fold serial dilution of standard DNA (10^6^ to 10^1^ copies/well). The number was converted to the number per milliliter of the wastewater sample. The number of SaV genomic copies has been quantified since April 2015. Since AstV, RVA, and RVC were detected without quantification, the amount of these viral genes was assessed using their real time PCR Ct values. Ct value is the number of cycles in real-time PCR when the PCR amplified product reaches a certain amount (threshold value). Ct values are inversely proportional to the amount of target nucleic acid in the sample.

### Quality control

As the internal control, pepper mild mottle virus (PMMoV) RNA was detected in the wastewater samples through real-time PCR using One Step PrimeScript™ III RT-qPCR Mix with UNG (Takara Bio) using QuantStudio5 (Applied Biosystems).^21^^–^^23^ The PMMoV is very abundant in wastewater.^21^^,^^22^ The PMMoV gene detection results were not used to calculate the efficiency of virus recovery but were used to check for substantial loss of the targeted viruses through comparison with previous data.^21^^,^^24^^,^^25^

### Number of gastroenteritis virus-positive samples

The number of gastroenteritis virus-positive specimens, reported based on the National Epidemiological Surveillance of Infectious Disease (NESID) program, was obtained information published by Chiba Prefecture.^26^^–^^31^ The same gastroenteritis viruses that we attempted to detect in wastewater samples were targeted. Sample collection and pathogen detection were performed according to the implementation manual of the NESID program.^6^^,^^32^

### Number of patients with infectious gastroenteritis

The number of pediatric patients with infectious gastroenteritis in each pediatric sentinel site was reported weekly to the Infectious Disease Surveillance Center of Chiba Prefecture in accordance with the NESID program.^6^ Infectious gastroenteritis is defined as abdominal pain, vomiting, and diarrhea with sudden onset. It is defined by the Act on the Prevention of Infectious Diseases and Medical Care for Patients with Infectious Diseases (the Infectious Diseases Control Law). There were 134 pediatric sentinel sites in Chiba Prefecture during the study period. The number of patients reported per pediatric sentinel site was published by Chiba Prefecture.^33^ Data collected from April 2014 to December 2019 were obtained from the official information portal of the Infectious Disease Surveillance Center of Chiba Prefecture and used for this study.

### Statistical analysis

“Imitating” cross-correlation was tested as follows. Spearman’s correlation coefficient rank test was performed between the number of NoV GI, NoV GII, and SaV genome copies in wastewater and number of reported patients per pediatric sentinel site in the week of the sampling day; 1 week, 2 weeks, 3 weeks, and 4 weeks before the sampling week; and 1 week, 2 weeks, 3 weeks, and 4 weeks after the sampling week, instead of only using the number of reported patients per pediatric sentinel site from the week of the wastewater sampling day. Stactcel4 (OMS Publishing Inc., Saitama, Japan) was used for statical analysis.

### Ethical approval

Ethics approval was exempt for this study because it uses non-human (wastewater-derived) data and publicly available databases.

## RESULTS

In the quality control evaluation, the number of PMMoV genome copies in the wastewater samples ranged from 3.58 to 4.81 log_10_ copies/mL. No seasonal differences were observed in PMMoV detection during the investigation period (data not shown). This result suggested that there was no substantial loss of viruses in the concentration of wastewater samples or any detrimental effects that could be attributed to PCR inhibitors present in wastewater.

**Figure 1.**
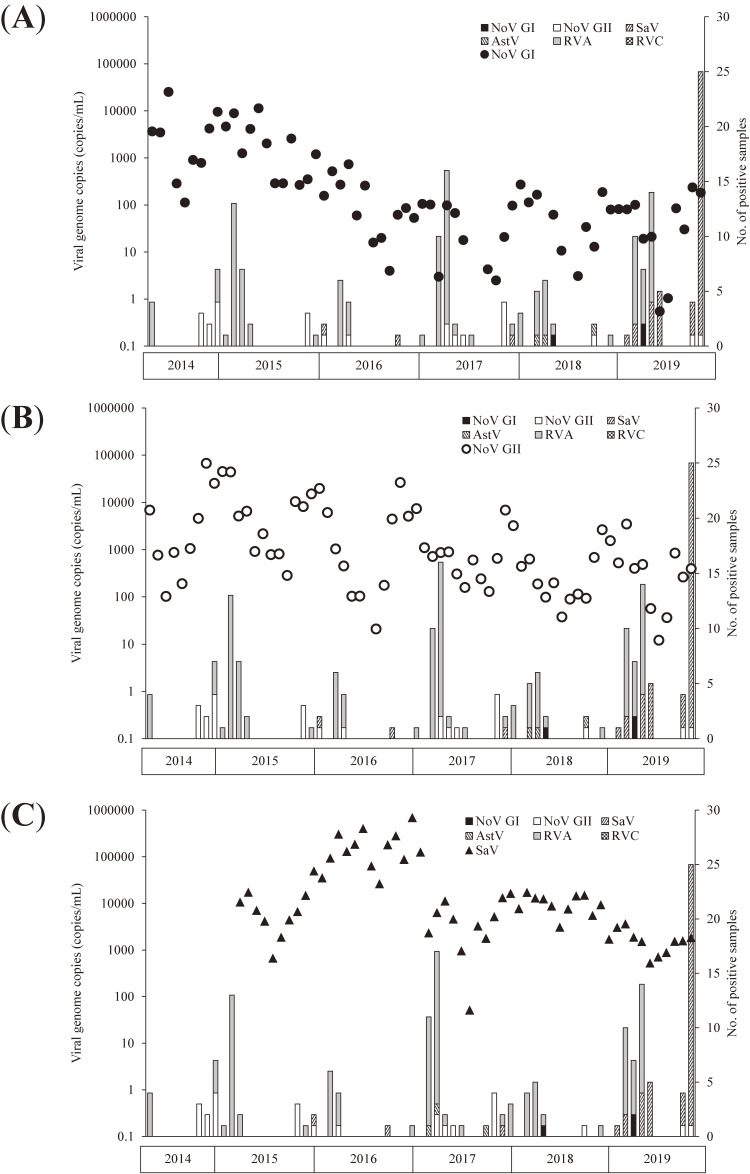
Genome copies of norovirus genotype I (NoV GI) (**A**), norovirus genotype II (NoV GII) (**B**), and sapovirus (SaV) (**C**) in wastewater and the number of gastroenteritis virus-positive samples reported through NESID.

**Figure 2.**
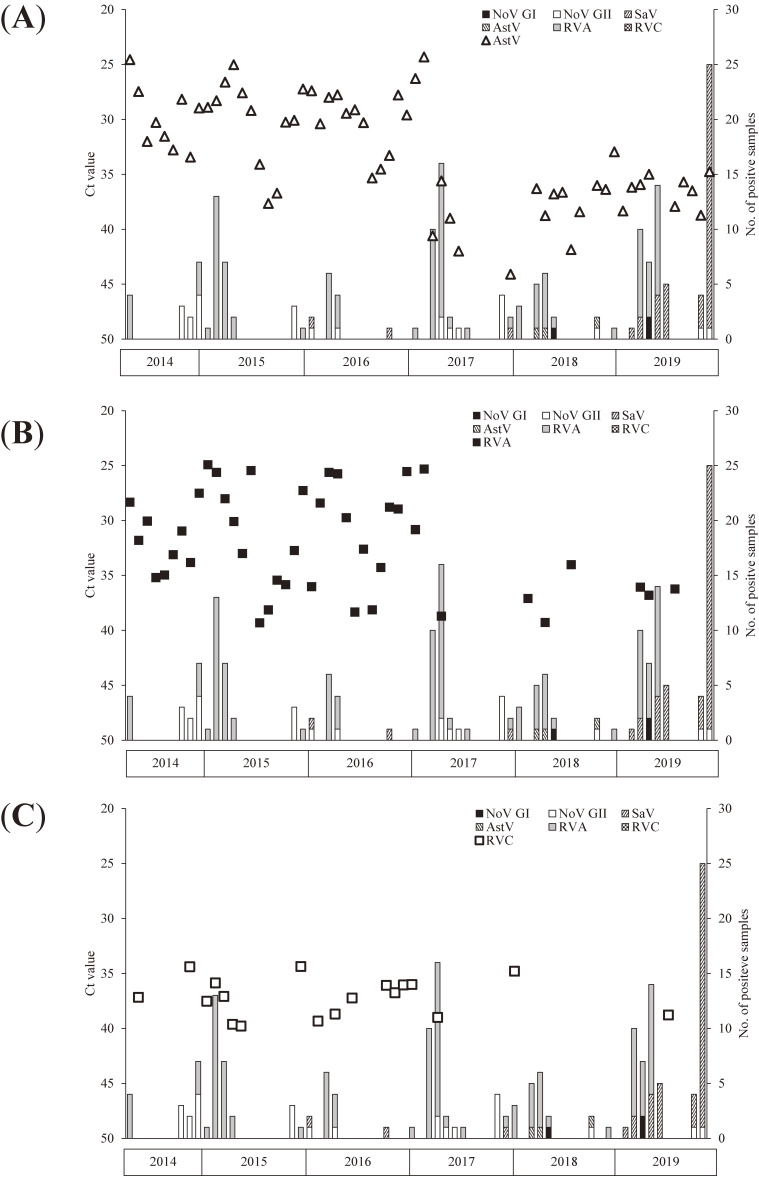
Cycle threshold (Ct) values of astrovirus (AstV) (**A**), rotavirus group A (RVA) (**B**), and rotavirus group C (RVC) (**C**) in wastewater and the number of gastroenteritis virus-positive samples reported through NESID. The vertical axis is inverted because a lower Ct value indicates a higher copy number.

**Figure 3.**
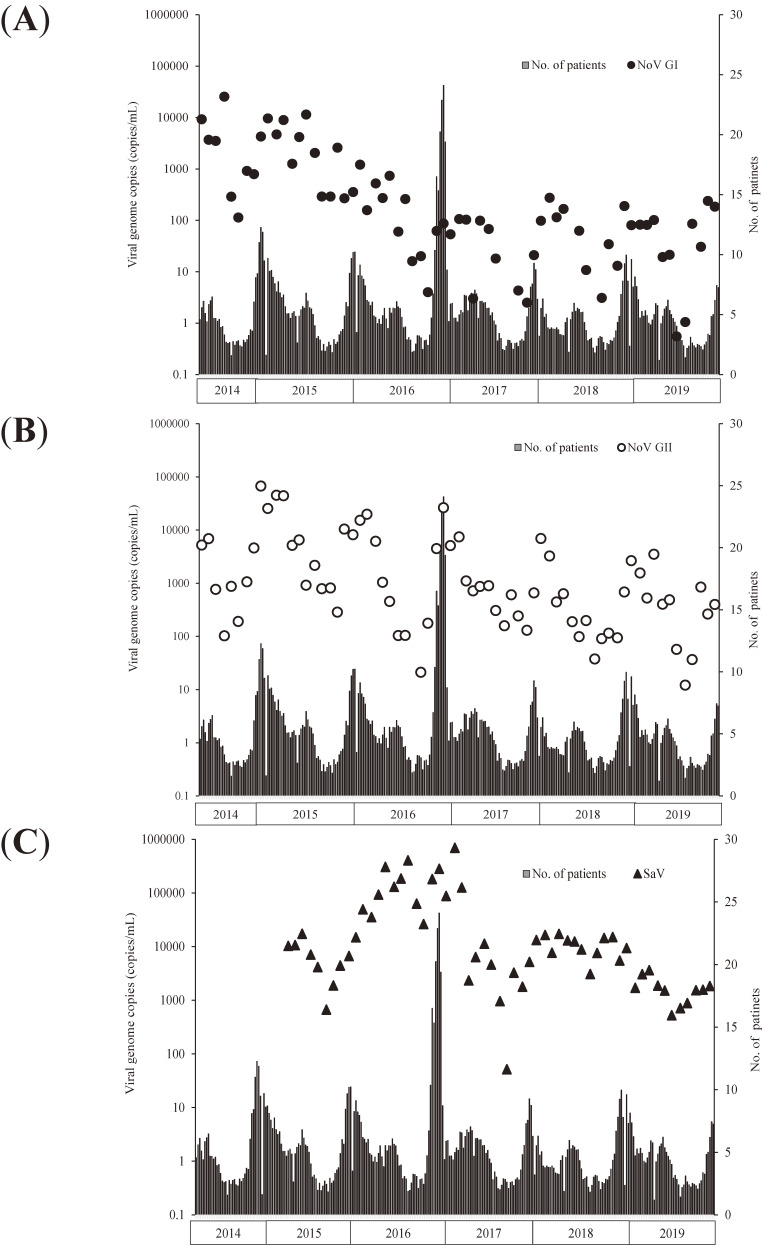
Trend in the detection of virus genome copies of norovirus genotype I (NoV GI) (**A**), norovirus genotype II (NoV GII) (**B**), and sapovirus (SaV) (**C**) in wastewater and number of reported patients with infectious gastroenteritis per pediatric sentinel site.

**Figure 4.**
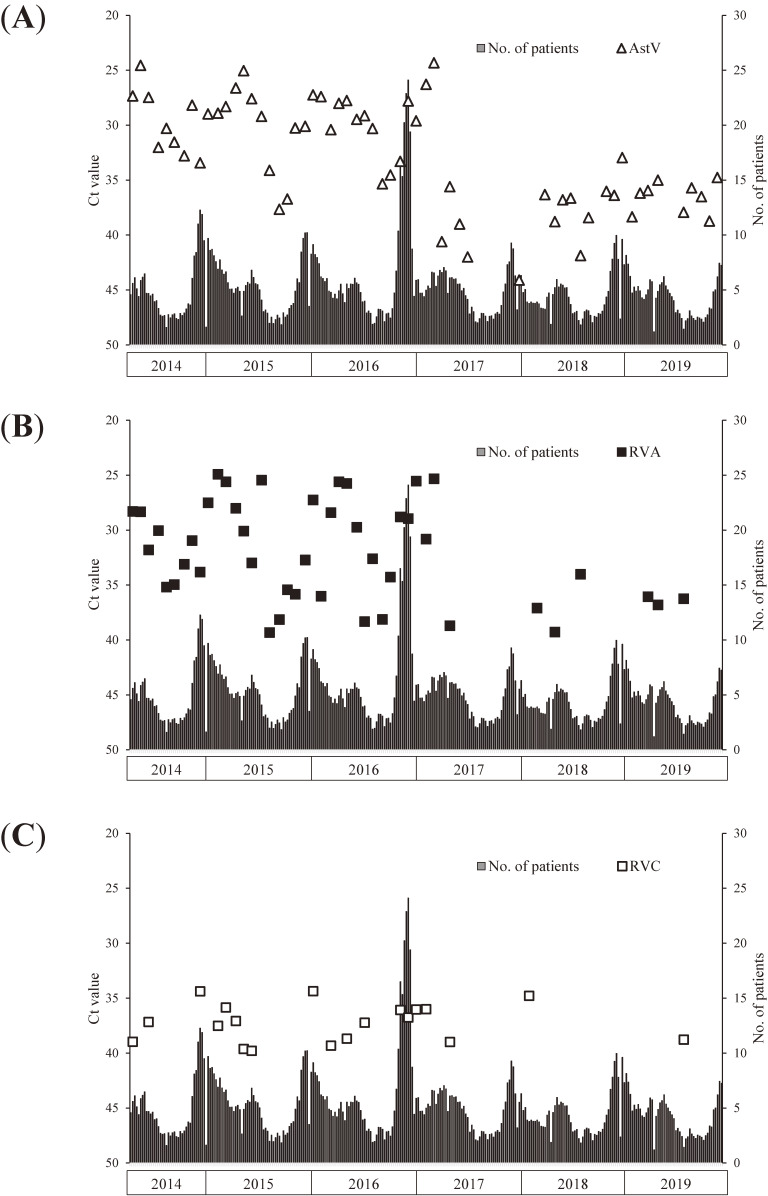
Trend in the cycle threshold (Ct) values of astrovirus (AstV) (**A**), rotavirus group A (RVA) (**B**), and rotavirus group C (RVC) (**C**) in wastewater and number of reported patients with infectious gastroenteritis per pediatric sentinel site. The vertical axis is inverted because a lower Ct value indicates a higher copy number.

NoV GI, NoV GII, and SaV were detected in the wastewater samples almost throughout the investigation period, whereas AstV, RVA, and RVC were intermittently detected (Figure 1, Figure 2, Figure 3, and Figure 4).

The viruses targeted in this study were detected in wastewater samples almost consistently throughout the study period. However, during certain periods, none of these six viruses were detected in the samples collected in the NESID program (Figure 1 and Figure 2). Of the number of gastroenteritis virus-positive samples reported through NESID, the number of RVA-positive samples was the highest. Contrarily, no samples were positive for RVC.

The relationship between the detection of viral genome copies in wastewater and number of patients per pediatric sentinel site is shown in Figure 3 and Figure 4. Among NoV GI, NoV GII, and SaV, there was a clear positive correlation most strongly between NoV GII detection and the number of reported patients per pediatric sentinel site in the week of the sampling day (Table 1). Moreover, the “imitating” cross-correlation test revealed an obvious positive correlation between NoV GII detection and the number of reported patients per pediatric sentinel site (Table 1).

**Table 1.** Correlation coefficients between the viral gene types detected in the monthly wastewater sample and the number of patients per pediatric sentinel site

Sample Timing At Sentinel Site	NoV GI		NoV GII		SaV	
	rs	*P* value^a^	rs	*P* value^a^	rs	*P* value^a^
4 weeks before ww	0.296	0.02	0.610	<0.001	0.385	0.004
3 weeks before ww	0.288	0.02	0.579	<0.001	0.347	0.009
2 weeks before ww	0.312	0.01	0.593	<0.001	0.349	0.009
1 week before ww	0.194	0.11	0.411	0.001	0.295	0.03
Week of ww sampling	0.324	0.008	0.625	<0.001	0.354	0.008
1 week after ww	0.329	0.006	0.656	<0.001	0.299	0.03
2 weeks after ww	0.234	0.05	0.540	<0.001	0.271	0.04
3 weeks after ww	0.095	0.44	0.378	0.002	0.333	0.01
4 weeks after ww	0.258	0.04	0.522	<0.001	0.275	0.04

NoV GI, norovirus genotype I; NoV GII, norovirus genotype II; rs, Spearman’s rank correlation coefficient; SaV, sapovirus; ww, wastewater.

^a^Two-sided test, 5% significance level.

## DISCUSSION

The number of pediatric patients with infectious gastroenteritis has been reported weekly by pediatric sentinel sites in Japan since the Infectious Disease Control Law was amended in 1999.^34^ We showed that gastroenteritis viruses, specifically, NoV GI, SaV, AstV, RVA, and RVC, were detected in wastewater even during periods when no gastroenteritis virus-positive samples were reported. The trend of the presence of these viruses in wastewater suggests that there are many asymptomatic infected child and adult patients infected. Furthermore, our results indicate that the reported trends in the number of patients per pediatric sentinel site are most reflected in the number of NoV GII gene copies detected in wastewater.

Our results indicate that gastroenteritis viruses can be detected in wastewater even during periods when gastroenteritis virus detection is not captured by the NESID program. Currently, pathogen detection in infectious gastroenteritis surveillance in Japan is based on the discretionary collection of specimens by pediatric sentinel sites.^6^ There has been a decreasing trend in the number of these samples in recent years.^7^ Therefore, it is difficult to properly identify the pathogenic viruses causing infectious gastroenteritis with the current surveillance system. The detection of viruses in wastewater indicates that there were cases of viral infection in the population served by the wastewater pumping station. It is known that RVA infections can cause encephalopathy,^35^ and SaV and AstV are known to cause infectious gastroenteritis in adults of all ages as well as in children.^36^^–^^38^ Even if these viruses are detected far less frequently than NoV GII in patients with infectious gastroenteritis, they should not be overlooked for public health reasons. It is possible to monitor and further analyze the viruses detected in wastewater to understand the prevalence of viruses in the population served by pumping stations.

NoV GII is passed in the stool of patients of all ages who are infected with the virus, which is how it flows into wastewater. The number of reported patients per pediatric sentinel site correlated most strongly with the number of NoV GII genome copies in wastewater. Therefore, the number of reported patients per pediatric sentinel site reflects that of all the cases of NoV GII-related gastroenteritis.

Adenoviruses and enteroviruses are also commonly detected as causative viruses of infectious gastroenteritis.^3^ We excluded adenovirus and enterovirus from this study. However, we considered that this exclusion did not seriously influence our findings. The detection of adenovirus and enterovirus in patients with infectious gastroenteritis is far less frequent than that of NoV GII.^39^^–^^41^ It is also known that NoV GII is the predominant virus detected in patients in the pediatric sentinel sites.^42^ This should have little influence on the present study, which examined the correlation between the number of reported patients and detection of viruses in wastewater.

We showed that NoV GII and other gastroenteritis viruses were detected in wastewater even during periods when no gastroenteritis virus-positive samples were reported. These results suggested that WBE could complement sentinel surveillance. Therefore, WBE could be a useful tool for surveillance of infectious gastroenteritis.
